# Choroidal sarcoid granuloma: a case report and review of the literature

**DOI:** 10.1186/s12348-022-00309-y

**Published:** 2022-09-29

**Authors:** Dany G. Hage, Charbel H. Wahab, Wajiha J. Kheir

**Affiliations:** 1 Department of Ophthalmology, American University of Beirut Medical Center, Beirut, Lebanon; 2 Tulane University School of Medicine, New Orleans, Louisiana USA

**Keywords:** Choroidal granuloma, Ocular sarcoidosis, Choroidal mass

## Abstract

**Background:**

Choroidal sarcoid granulomas are often diagnosed in patients without a prior history of sarcoidosis. They are often mistaken for choroidal metastasis, choroidal nevi, amelanotic choroidal melanomas, and uveal lymphomas; however, are easily treatable when accurately identified.

**Observations:**

We searched PubMed, Medline, and Scopus for English-Language case reports published before September 2021. Additionally, we presented a case of a 45-year-old woman with a right-sided amelanotic choroidal mass whose diagnosis was delayed by a COVID-19 infection. Of the 26 cases reported in the literature, 46% were female, 38% were African American, and 19% had bilateral involvement. There was a mean age of 42.15 years and a mean follow-up period of 27 months. The most common complaint was of a progressive, painless blurring of vision, and only five patients had been previously diagnosed with sarcoidosis. The choroidal granulomas were typically described as yellow lesions, single or multiple, found temporal to or at the macula. Most patients were administered steroids, with 69% receiving them systemically, 5% topically, and 8% locally with a triamcinolone injection. All patients reported symptomatic improvement at their final follow-up with resolution of the mass in 65% of patients and improved visual acuity in 76%.

**Conclusion:**

Primary testing including fundoscopy, fluorescein angiography, fundus autofluorescence, A/B-scan, and OCT are useful for diagnosis, differentiation from other choroidal lesions, and monitoring treatment response. Steroids are a mainstay of treatment for sarcoidosis and are effective at treating choroidal granulomas. Therefore, early recognition and diagnosis of choroidal granulomas is imperative as treatment can be curative and sight-sparing.

## Introduction

Sarcoidosis is a chronic inflammatory granulomatous disease that can affect multiple major organ systems including the eye and its surrounding structures [1]. The wide variability of clinical manifestations has posed diagnostic challenges that make it difficult to ascertain the true prevalence of the disease [2]. It is currently estimated that 13–79% of those affected by sarcoidosis will develop ocular manifestations [2–5]. Ocular symptoms are often the initial notable finding in 20–30% of cases, with uveitis (30–70%) and conjunctival nodules (40%) being the most encountered [3, 6, 7]. Additionally, studies by Rothova et al. and Evan et al. demonstrated that females (56% versus 23%) and African Americans are more likely to develop ocular involvement [2, 7, 8].

Uveitis is an inflammatory process that affects the uveal tissues - iris, ciliary body, choroid – and surrounding structures – anterior chamber, retina, vitreous humor [2]. It is often identified on slit lamp or fundus examination and classified as anterior, intermediate, posterior, or panuveitis [2]. When associated with sarcoidosis, uveitis can be further categorized based on the presence of granulomatous inflammation, and a study of 112 eyes by Dana et al. noted a prevalence of granulomatous inflammation in 81% of those with sarcoid uveitis [2, 9]. Choroidal inflammation can lead to the development of choroidal granulomas and symptoms differ based on the location of the lesion (central versus peripheral) [2]. Furthermore, the granulomas may vary in size and be unifocal or multifocal [2, 10, 11]. They can also lead to choroidal neovascularization and exudative retinal detachments [2, 12].

This article summarizes all published case reports documenting choroidal sarcoid granulomas in the English language from December 1982 to July 2021 and presents a unique case of a choroidal granuloma in a patient with previously undiagnosed sarcoidosis that was initially mistaken for malignancy.

## Methodology

A literature review accessing PubMed, Medline, and Scopus databases was performed in September 2021. The keywords “choroid,” “sarcoid,” “sarcoidosis” and “granuloma” were used to gather all peer-reviewed case reports published in the literature. Non-English case reports were excluded from the review. We used the PRISMA and CARE checklists when writing our report [13, 14].

### Case report

A 45-year-old previously healthy female presented to the Ophthalmology Specialty Clinics at the American University of Beirut Medical Center with a referral diagnosis of a suspicious choroidal lesion associated with subretinal fluid in the right eye. The patient had no personal or family history of cancer. The patient was a current everyday hubble-bubble smoker but did not consume alcohol or use recreational drugs.

**Fig. 1:**
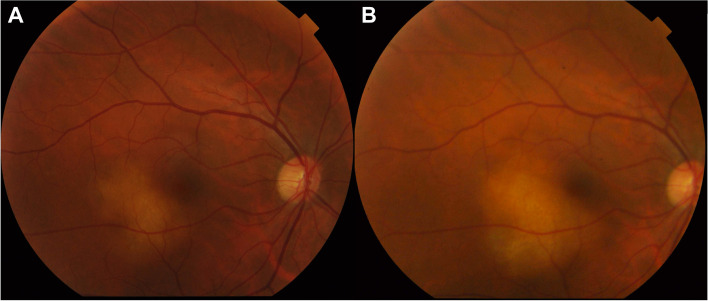
**A** Right fundus photo showing a yellow elevated choroidal lesion inferior temporal to the fovea with 2 × 3 mm basal diameter and overlying subretinal fluid. **B** shows growth of the lesion on 3-month follow-up

**Fig. 2:**
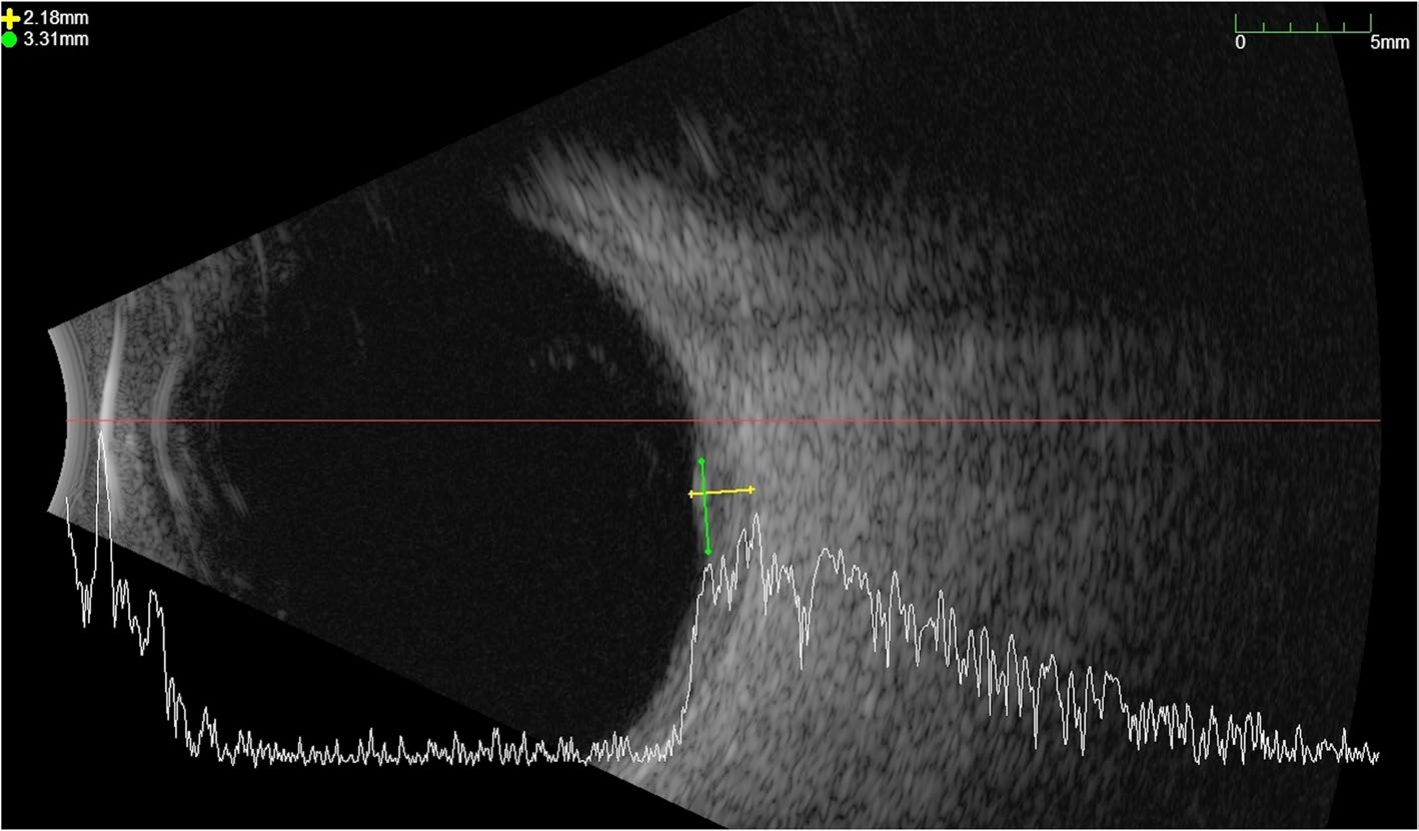
B Scan showing a shallow dome-shaped, regularly structured, hyperechoic lesion of the infratemporal choroid with a maximal thickness measured of 2.18 mm and a diameter of 3.21 mm. No distinct extrascleral extension was noted

**Fig. 3:**
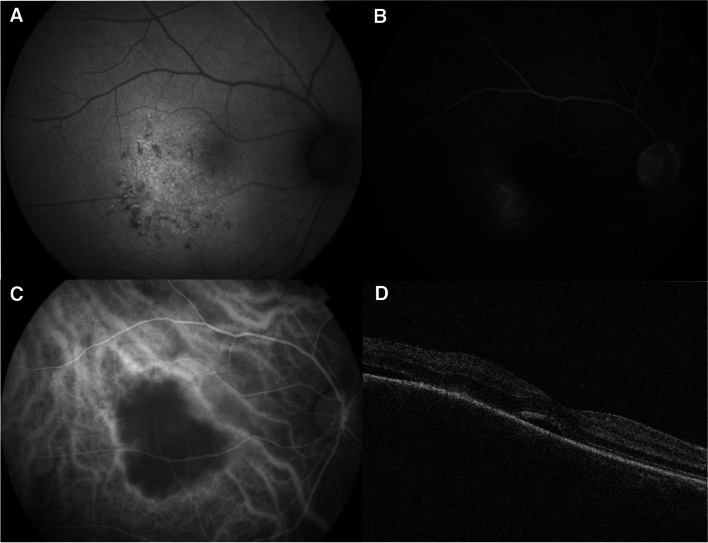
(**A**) Fundus autofluorescence of the right eye showing a hyperautoflourescence with hypoautofluorescence stippling at the lesion. On Fluorescein angiography (**B**), there is late diffuse staining of the entire lesion. On Indocyanine green angiography (**C**), the lesion exhibits hypocyanescence at all phases. (**D**) Optical coherence tomography (OCT) of the right eye shows choroidal elevation and subretinal fluid at the macula

Best-corrected visual acuity was 20/25 in the right eye and 20/20 in the left eye. The anterior segment slit-lamp examination was unremarkable except for trace nuclear sclerosis in the right eye. Funduscopic examination demonstrated a quiet, acellular vitreous in both eyes with a yellow elevated choroidal lesion infra-temporal to the fovea of the right eye with a 2 × 3 mm basal diameter and overlying subretinal fluid **(**Fig. 1A). B-scan ultrasonography showed a dome-shaped, regularly structured, hyperechoic lesion of the choroid. The maximal thickness measured 2.18 mm, and no distinct extrascleral extension was noted (Fig. 2). Fundus autofluorescence (AF) of the right eye showed hyperautoflourescence with hypoautoflourescent stippling at the lesion **(**Fig. 3A). Fluorescein angiography (FA) showed normal retinal vasculature and a late diffuse staining of the entire lesion (Fig. 3B), whereas the indocyanine green angiography (ICG) showed an early hypocyanescence of the lesion that persisted into late into angiography (Fig. 3C). On optical coherence tomography (OCT) there was a right choroidal elevation with associated subretinal hyperreflective material and subretinal fluid (Fig. 3D).

Our differential diagnosis included amelanotic choroidal nevus/melanoma, hemangioma, uveal lymphoma, metastasis, and masqueraders. Given the results of the ophthalmic imaging, we were most suspicious of uveal lymphoma, metastasis, or a granulomatous process.

The patient was sent for complete oncologic screening examinations including mammography, chest, abdomen, and pelvis computerized tomography (CT) scan, and brain and orbit magnetic resonance imaging (MRI). All were non-revealing with no evidence of malignancy except for the chest CT scan showing few scattered subcentimetric lymph nodes in prevascular and bilateral hilar spaces. The decision was made for close follow-up considering the difficulty, low yield potential and vision threatening risk of a biopsy of the lesion. After an increase in subretinal fluid (SRF) and slight increase in diameter was noted on the 3-month follow-up **(**Fig. 1B), an oncologist was consulted. The recommended positron emission tomography (PET) scan showed concerning fluorodeoxyglucose (FDG) avid thoracic, abdominal, and pelvic lymphadenopathy. One day after the scan, the patient developed fever and upper respiratory tract symptoms and tested positive for COVID-19. Biopsy of the lymph nodes was deferred as oncology suspected the viral infection may have been the cause of her lymphadenopathy.

**Fig. 4:**
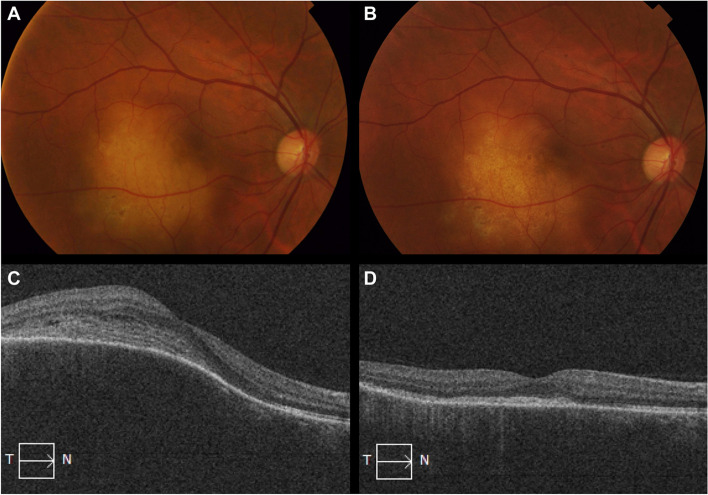
Right fundus photo and OCT of the choroidal lesion before (**A** & **C**) treatment and after (**B** & **D**) treatment with oral steroids. There is complete regression of the choroidal lesion with residual chorioretinal atrophy (**B**) with corresponding flattening of choroidal elevation on OCT and loss of ellipsoid zone at the area of atrophy (**D**)

A repeat PET-CT scan at 3 months showed persistent lymphadenopathy. Clinically, the patient’s vision had worsened to 20/200 and the choroidal lesion had continued to increase in size with a largest basal diameter of 6.81 mm and a maximal thickness of 3.01 mm. An endobronchial ultrasound guided biopsy revealed a non-necrotizing granuloma suggestive of sarcoidosis. Systemic steroid therapy was initiated resulting in an improvement in vision to 20/60 and complete regression of the choroidal lesion with residual chorioretinal atrophy **(**Fig. 4**)**.

### Review of the literature

A summary of the 26 published cases of sarcoid granulomas is presented in Table 1. Regarding patient demographics, 46% of patients were female (12/26), and the mean age at diagnosis was 42.15 (range: 10–67). There was only one pediatric patient among the cases who was 10 years old at the time of diagnosis [21]. Of the 26 cases, 16 reported racial demographics with 38% of patients being African American (6/16) [17, 20, 23, 34, 36], 38% Caucasian (6/16) [21, 24, 28, 32, 35, 36], and 25% Asian (4/16) [26, 27, 29, 33]. Among the 26 patients, 30 total eyes were affected, with 19% of patients having bilateral involvement (5/26) [16, 18, 25, 32], and the majority having unilateral involvement [42% right eye (11/26) [15, 17, 20–22, 24, 26, 31, 34, 36], 38% left eye (10/26) [18, 19, 23, 27–30, 33, 35]]. The mean follow-up period was 27 months (median: 12, range: 1–216). The most common visual complaint was a progressive painless blurring of vision (21/26) [15, 17, 18, 20, 21, 23–26, 28, 29, 31–34]. Other presentations included floaters (4/26) [15, 18, 20], eye pain (2/26) [23, 32], headaches (2/26) [17, 32], and those that were asymptomatic (5/26) [16, 19, 22, 27, 30].

**Table 1:** Review of cases of choroidal sarcoid granuloma

Year	Author	Sex	Age (yrs.)	Race	Eye	Presenting Symptoms	F/U (mo.)	Treatment	Outcome
2010	Verma et al. [15]	M	46	N/A	OD	Painless, gradual loss of vision in the right eye with floaters for 20 days	1	Systemic steroids	Reduction in mass size Improvement in visual acuity (VA)
2020	Ung et al. [16]	M	55	N/A	OU	Asymptomatic	12	No treatment Tapering of Nivolumab	Stabilization in the size of the mass
1982	Marcus et al. [17]	F	19	African American	OD	Blurred vision in the right eye for 2 months	6	Systemic steroids	Resolution of mass VA of 20/20
1982	Marcus et al. [17]	F	31	African American	OD	Decreased vision in the right eye for 1 week with right sided headaches	12	Systemic steroids	Resolution of mass VA of 20/20
2009	Doycheva et al. [18]	F	49	N/A	OU	Visual disturbances for 2 months	48	Systemic and topical steroids Tapering of INF-a	Resolution of mass Improvement of VA
2009	Doycheva et al. [18]	M	37	N/A	OU	Floaters and decreased vision	60	Topical steroids Tapering of INF-a	Resolution of mass VA of 20/20
2009	Doycheva et al. [18]	F	65	N/A	OS	Visual disturbances and floaters for 8 days	72	Topical steroids Tapering of INF-a	Resolution of mass VA of 20/20
2017	Turkoglu et al. [19]	M	66	N/A	OS	Asymptomatic	2	Systemic steroids	Resolution of mass
2020	Schönbach et al. [20]	M	67	African American	OD	Progressive decreased vision and floaters in the right eye	3.5	Topical Cyclopentolate and steroids Triamcinolone Injection Systemic steroids	Resolution of mass VA of 20/20
2021	Pichi et al. [21]	F	10	Caucasian / Middle Eastern	OD	Blurry vision in the right eye	N/A	Afilbercept Systemic steroids	Initially worsened with Afilbercept but improved after steroids Resolution of mass Improvement of VA
2017	Stefater et al. [22]	F	55	N/A	OD	Asymptomatic	216	N/A	Gradual increase in mass size over time with development of smaller lesions VA of 20/20
2018	Knickelbein et al. [23]	F	25	African American	OS	Bilateral red sore eyes for 3 months Decreased vision in the left eye for 2 months	30	Systemic steroids Cyclosporine Mycophenolate Infliximab	Resolution of mass Improvement of VA
2021	Armbrust et al. [24]	M	54	Caucasian	OD	Blurry vision in the right eye over 2–3 months	12	Systemic steroids Adalimumab	Residual lesion present
2013	Chen et al. [25]	M	50	N/A	OU	Vision loss with floaters in the right eye for 3 years Decreased vision in the left eye for 11 months	6	Combination steroid and immunosuppressive drugs	Improvement observed
2017	Ishihara et al. [26]	M	38	Asian	OD	Blurry vision in the right eye	6	Systemic steroids Triamcinolone injection	Resolution of mass
2021	Kita et al. [27]	M	36	Asian	OS	Asymptomatic	N/A	Triamcinolone injection	Resolution of mass VA of 20/20
1983	Olk et al. [28]	F	25	Caucasian	OS	Central blurring in the left eye for 3 days	12	Systemic steroids	Resolution of mass VA of 20/20
2021	Kobayashi et al. [29]	M	38	Asian	OS	Decreased visual acuity in the left eye	12	Systemic steroids Triamcinolone injection	Reduction in mass size Improvement of VA
2014	Khatib et al. [30]	F	52	N/A	OS	Asymptomatic	5	Triamcinolone injection	Resolution of mass VA of 20/20
2013	Kumar et al. [31]	F	35	N/A	OD	Painless decreased vision in the right eye	8	Triamcinolone injection Topical steroids	Resolution of mass Improvement of VA
2017	Pandya et al. [32]	M	30	Caucasian / Middle Eastern	OU	Progressively worsening headaches for 6 weeks with a 2-week history of bilateral ocular pain, redness, photophobia, and blurred vision	N/A	Systemic steroids Methotrexate Mycophenolate Infliximab Triamcinolone injection	VA of 20/20 Patient required bilateral Trabeculectomy for elevated IOP
2005	Chan et al. [33]	M	29	Asian	OS	Left paracentral visual field loss for 2 weeks	48	Systemic steroids Triamcinolone injection	Reduction in mass size Improvement of VA
2013	Modi et al. [34]	F	63	African American	OD	Painless decrease in vision of the right eye with metamorphopsia for 3 months	12	Systemic steroids	Resolution of mass Improvement of VA
1992	Tingey et al. [35]	M	26	Caucasian	OS	Blurred vision in the left eye for 8 weeks	13	Systemic steroids	Resolution of mass Worsening of VA
1984	Campo et al. [36]	M	33	African American	OD	Two months of blurred vision in the right eye	10	Systemic steroids	Resolution of mass Improvement of VA
1984	Campo et al. [36]	F	62	Caucasian	OS	Blurred vision in the left eye for 1 week	8	Systemic steroids	Reduction in mass size Improvement of VA
2022	Hage et al	F	45	Caucasian /Middle Eastern	OD	Asymptomatic	9	Systemic steroids	Resolution of mass Worsening of VA compared to baseline

#### Past medical history

Five patients had been diagnosed with sarcoidosis before developing choroidal granulomas [17, 19, 20, 27, 34], one patient had a history of psoriatic arthritis with sacroiliitis [24], two patients had a prior diagnosis of bilateral granulomatosis panuveitis [23, 26], and one patient had been diagnosed with bilateral granulomatous iritis 2.5 years prior to the development of the choroidal granuloma [26, 36]. Interferon-alpha (INF-a) use was found in 3 patients (2 female, 1 male) that were undergoing treatment for chronic Hepatitis C [18]. Two men reported a prior history of prostate cancer, one treated with radiation therapy [20] and the other with radical prostatectomy [19]. Two patients (1 female, 1 male) had a prior history of melanoma, with one patient undergoing Nivolumab therapy for metastasis [16] and the other having 5 prior excisions for dermal melanoma [22].

#### Examination findings and imaging

A summary of all the examination and imaging findings of the 26 cases is presented in Table 2. Fundoscopy was used in all 26 of the cases reviewed. Of the 26, there were single granulomas in 54% (14/26) [15, 17, 19, 21–24, 27–29, 31, 35, 36] and multiple in 46% (12/26) [16, 18, 20, 25, 26, 30, 32–34, 36]. Most lesions were yellow (13/26) [16, 17, 19–21, 23, 25, 26, 32, 35, 36] and located at, or temporal to the macula (9/26) [17, 21, 23, 27, 29, 31, 35, 36]. Some lesions were described as elevated (9/26) [15, 17, 21, 23, 32, 34–36], polypoid (1/26) [22], round (1/26) [27], white (3/26) [18, 27, 29], and creamy (3/26) [16, 30, 34]. Other notable locations include the posterior segment (4/26) [20, 22, 32, 34], optic disc (2/26) [28, 30], fundus (4/26) [18, 19, 25], fovea (1/26) [36], and arcade (3/26) [15, 16, 26]. Additionally, retinal detachments (6/26) [17, 20, 28, 36] and a choroidal detachment (1/26) [20] were observed.

**Table 2:** Summary of tumor descriptions and imaging results

Year	Author	Tumor Description	B/A Scan Ultrasound	Fluorescein Angiography	Fundus Autofluorescence	OCT	Indocyanine Green
2010	Verma et al. [15]	Elevated mass along the supertemporal arcade, with minimal subretinal fluid surrounding it	Localized retinochoroidal elevated mass over the posterior pole with high surface reflectivity, low internal reflectivity, and surrounding shallow subretinal fluid	Diffuse hyperfluorescence with retinal pigment epithelial window defects over the lesion			
2020	Ung et al. [16]	Creamy yellow choroidal lesions, one inferotemporal to the macula, one supertemporal to the macula, and one inferior to the arcade	No posterior elevation of the lesion				
1982	Marcus et al. [17]	Moderately elevated, yellowish choroidal mass centered in the macula with mild pigment dispersion					
1982	Marcus et al. [17]	Yellowish choroidal lesion temporal to the macula, impinging upon the fovea, with a large posterior neurosensory detachment		Initial hypofluorescence with faint staining of the chorioretinal lesion by the venous phase			
2009	Doycheva et al. [18]	Small white chorioretinal lesions with pigment epithelial alterations in the midperiphery of the retina and granulomas at the fundus					
2009	Doycheva et al. [18]	Small chorioretinal granulomas at the fundus around the optic disc and in the midperiphery of the retina					
2009	Doycheva et al. [18]	Small chorioretinal granulomas in the midperiphery of the retina					
2017	Turkoglu et al. [19]	Yellow 2 mm choroidal mass in the temporal macular region, with a 1 mm chorioretinal scar superior to the optic disc				Homogenous hyporeflective lesion in the choroid, 246 μm, with intact overlying retinal pigment epithelium and compression of the choroidal vascular structures	
2020	Schönbach et al. [20]	Yellowish choroidal lesions in the posterior pole		Scattered hyperfluorescent spots within the macula with mild leakage		Subretinal fluid and choroidal corrugations (2 weeks after treatment)	
2021	Pichi et al. [21]	Elevated yellowish lesion at the center of the macula surrounded by a ring of subretinal fluid and blood				Hyperreflective homogenous material in the choroid obliterating the inner and outer choroidal vasculature and eroding through the Bruch membrane, with associated subretinal fluid and intraretinal cysts	
2017	Stefater et al. [22]	Minimally elevated, nonpigmented choroidal lesion with polypoidal borders at the posterior segment	No elevation				
2018	Knickelbein et al. [23]	Elevated yellow choroidal mass temporal to the macula of the left eye		Right eye with disc hyperfluorescence, blockage in the areas of hemorrhage, and diffuse small vessel leakage. Left eye with early hyperfluorescence of the optic nerve that was maintained into late frames, diffuse small vessel leakage, and early hypofluorescence with late speckled hyperfluorescence of the large temporal choroidal mass	Right eye with hypoautofluorescence in the areas of hemorrhage Left eye with small speckled hypoautofluorescence overlying the choroidal mass	Right macula with subretinal fluid nasally adjacent to the nerve and retinal folds Left macula with large intraretinal cystic fluid pockets nasally and temporally, subretinal fluid nasally adjacent to the nerve, foveal detachment with nasal and temporal choroidal elevation	
2021	Armbrust et al. [24]	N/A				EDI-OCT: Isolated peripapillary choroidal granuloma with associated optic disc edema	
2013	Chen et al. [25]	Extensive, confluent, subretinal, yellowish white infiltrates throughout the fundus with peripapillary atrophy					
2017	Ishihara et al. [26]	Right eye with multifocal yellowish-white lesions in the peripapillary region and the arcade and retinal periphlebitis with small perivascular nodules in the periphery Left eye with chorioretinal atrophic lesions in the periphery				Homogenous hyporeflectivity with thinning of the overlying choriocapillaris, with associated subretinal fluid adjacent to the peripapillary choroidal lesion	
2021	Kita et al. [27]	Round, white, 1.5 DD choroidal mass located 1 DD superior-temporal to the macula	Intrachoroidal mass lesion noted without calcification	Hypofluorescence during the early phase, with late leakage		Dome shaped elevated of the choroid with effacement of the choroidal vessels, and subretinal fluid accumulation over the mass and macula	Hypofluorescence persistent throughout the late phase of the angiogram
1983	Olk et al. [28]	Slightly depigmented choroidal lesion, 1 × 2 DD, in the papillomacular bundle adjacent to the optic nerve	Elevation of the neurosensory retina with fluid in the extrascleral space adjacent to the temporal aspect of the optic nerve	Multiple pinpoint areas of hyperfluorescence on the surface of the lesion, with late diffuse staining of the entire lesion with collection of dye under the neurosensory detachment			
2021	Kobayashi et al. [29]	Large white protruding lesion of 10 × 8 PD slightly temporal to the macular region	High internal reflectivity	Left eye with punctate fluorescein leakage at the early phase, with tissue staining and fluorescein pooling from the middle to late phase Right eye with slight fluorescein leakage in the peripheral region		Protruding lesion with a homogenous shadow on the choroid and under the retina, complicated by an exudative retinal detachment	Low fluorescence and filling delay in the area corresponding to the mass
2014	Khatib et al. [30]	Multiple cream-colored choroidal lesions involving the optic disc					
2013	Kumar et al. [31]	Solitary choroidal granuloma temporal to the macula, with disc edema and vitreous snowballs inferiorly	Choroidal granuloma confirmation			Choroidal elevation with intraretinal and subretinal fluid	
2017	Pandya et al. [32]	Multiple elevated, pale yellow choroidal lesions, consistent with granulomas, predominantly in the posterior pole of both eyes, and disk hyperemia, swelling, and a right partial macular scar		Early-phase hypofluorescence and late-phase hyperfluorescence of the choroidal granulomas with disk hyperfluorescence			
2005	Chan et al. [33]	Retinal and choroidal granulomas, with dilated inferotemporal retinal vein with loops, and subretinal exudates at the macula		Periphlebitis, staining of the inflammatory masses, and subretinal leakage			
2013	Modi et al. [34]	Multiple deep, focal, elevated, creamy lesions at the posterior segment and diffuse peripapillary focal creamy lesions obscuring the disc margins with adjacent subretinal fluid				Homogenous hyporeflective lesion, with thinning of the overlying and surrounding uninvolved choroidal architecture, and focal elevation of the retinal pigment epithelium with shadowing deep to the lesion and subretinal fluid	Disc leakage and hypofluorescence
1992	Tingey et al. [35]	Slightly elevated, pale yellow choroidal mass with overlying subretinal fluid involving the macula	1.4 mm choroidal mass with serous retinal detachment and high internal reflectivity.	Early hypofluorescence with later hyerfluorescence and leakage of dye into the subretinal space			
1984	Campo et al. [36]	Yellow-white elevated choroidal tumefaction, 2.5 DD, involving the right macula Left fundus with a white 1 DD scar above the superior arcade	2 mm of elevation, medium internal reflectivity	Initial relative hyperfluorescence with progressive dye accumulation in the late phase			
1984	Campo et al. [36]	Pale yellow choroidal tumefactions, 2 DD, above the fovea with small flecks of blood on the lower border and a shallow serous elevation of the retina extending inferiorly to involve the fovea Temporally similar choroidal mass, 1.5 DD, without serious retinal detachment. Pale grey choroidal thickening, 0.5 DD, above the fovea of the right eye with a small red center with a small red center.	Internal tissue characterization not possible	Diffuse hyperfluorescence with focal pigment epithelial staining			
2022	Hage et al	Yellow elevated choroidal lesion, 2x3x2.18 mm in the posterior pole	Dome-shaped, regularly structured, hyperechoic	Late diffuse staining	hyperautoflourescence with hypoautoflourescent stippling	Choroidal elevation with associated subretinal hyperreflective material and subretinal fluid	Hypocyanescence throughout angiography

Fluorescein angiography (FA) was used in 12 cases (46%) and demonstrated hyperfluorescence in 5 cases [15, 20, 23, 28, 36], early phase hypofluorescence with late-phase hyperfluorescence in 4 cases [23, 32, 35, 36], hypofluorescence in 2 cases [17, 27], punctate leakages in 1 case [29], subretinal leakages in 2 cases [33, 35] and periphlebitis in 1 case [33].

Fundus autofluorescence (AF) was used in 2 cases (8%) and showed hypoautofluorescence in areas of hemorrhage [23], speckled hypoautofluorescence overlying the lesion [23], and hypofluorescence and disc leakage near sites of the lesions [34].

Ultrasonography (A/B scan) was used in 9 cases (35%) and identified the presence of elevation in 4 cases [15, 28, 35, 36] and high internal reflectivity in 2 cases [29, 35], as well as the absence of elevation in 2 cases [16, 22] and the presence of calcification in 1 case [27]. It also noted subretinal fluid in 1 case [15], a retinal detachment in another [35].

Ocular coherence tomography (OCT) was used in 10 (38%) cases and was notable for a homogenous hyporeflective lesion in 1 case [19], a homogenous hyperreflective lesion in 3 cases with thinning of the overlying choroidal architecture in 2 of those cases [21, 26, 34], subretinal fluid in 8 cases [19–21, 23, 26, 27, 31, 34], an exudative retinal detachment in 1 case [29], intraretinal cysts in 1 case [21], optic disc edema in 1 case [24], and a dome-shaped elevation of the choroid with effacement of the vessels in 1 case [27].

Indocyanine green (ICG) was used in 3 cases (12%) and showed hypocyanescence through the late phase of the angiogram in 1 case [27], low hypercyanescence with a filling delay in the area corresponding to the mass in 1 case [29], and low hypocyanescence with disc leakage in the last case [34].

Additional systemic investigations revealed lymphadenopathy on imaging in 54% of cases (14/26) [15–18, 21, 23, 24, 28, 29, 33–36], elevated ACE levels in 38% of cases (10/26) [15, 18, 26, 28, 30–32, 35, 36], and noncaseating granulomas on biopsy in 69% of cases (18/26) [17, 22–28, 30, 32–36]. Of the 18 cases that collected a biopsy, 61% (11/18) were from lymph nodes [17, 22, 24, 27–29, 33, 35, 36], 28% (5/18) from skin [26, 27, 30, 32, 34], 6% (1/18) from the choroid [25], 11% (2/18) were bronchial [23, 27], and 6% (1/18) did not specify the location [16].

### Treatment and outcomes

Of the 26 cases, 18 (69%) received systemic steroids [15, 17–21, 23–26, 28, 29, 32–36], 5 (19%) received topical steroids [18, 20, 31], 8 (31%) received locally administered steroids in the form of a triamcinolone injection [20, 26, 27, 29–33], 4 (15%) received immunosuppressive agents [23–25, 32], and 1 (4%) received a topical muscarinic antagonist [20]. Two patients (8%) did not receive any treatment, however, of the two, the patient that was on Nivolumab had it tapered [16, 22]. All 26 patients had symptomatic improvement by their final follow up with a complete resolution of the choroidal mass in 65% of patients (17/26) [17–21, 23, 26–28, 30, 31, 34–36], and regression and stabilization of the mass in 31% of patients (9/26) [15, 16, 22, 24, 25, 29, 32, 33, 36]. There was a visual acuity reporting of 20/20 Snellen equivalent in 38% of cases (10/26) [17, 18, 20, 22, 27, 28, 30, 32] at final follow-up and notable improvement in 38% of cases (10/26) [15, 18, 21, 23, 29, 31, 33, 34, 36] after treatment. Only 1 case reported a gradual worsening in the size of the granuloma, however, the patient had a visual acuity of 20/20 and was observed over the course of 18 years without receiving any treatment [22].

## Discussion

In our case, the choroidal granuloma was the only clinical manifestation of sarcoidosis, which was discovered after systemic screening for neoplasms was performed. Diagnosis was delayed by a COVID-19 infection which confused the clinical presentation. Of the cases reviewed, only 5 patients had a sarcoidosis diagnosis prior to the identification of the choroidal granuloma [17, 19, 20, 27, 34]. Similarly, to our case, the choroidal granuloma was the initial manifestation of sarcoidosis in the remaining cases we reviewed. This underscores the masquerading quality of an isolated choroidal granuloma, posing a unique diagnostic challenge to clinicians. Therefore, thoroughly investigating the full differential diagnosis of a choroidal lesion – summarized in Table 3 is an important exercise, as there may be an underlying neoplastic process.

**Table 3:** Characteristics of the differential diagnosis to a choroidal sarcoid granuloma

Differential	Fundoscopy	Ultrasound	FA	AF	OCT	ICG
Metastasis	Yellow subretinal mass posterior to the equator with subretinal fluid Alternative: Orange mass or brown, grey lesions	Echo-dense lesions (B-scan) with high internal reflectivity (A-scan)	Early hypofluorescence in the arterial phase and late hyperfluorescence in the venous phase	Areas of hyperautofluorescence corresponding to subretinal fluid and lipofuscin (scattered clumps of brown pigment)	Undulating surface overlying the metastasis and thickening of the retinal pigment along areas of subretinal fluid	Hypocyanescence at all stages
Amelanotic Melanoma	Flat, or slightly elevated yellow white lesions, with poorly defined margins and often associated with drusen, prominent vascularity, subretinal fluid, a serous retinal detachment, and lipofuscin with an orange or golden-brown appearance	Acoustically hollow lesions (B-scan) with low internal reflectivity (A-scan)	Double circulation, extensive leakage with progressive fluorescence, late staining of the lesions, and multiple areas of pinpoint leaks affecting the retinal pigment epithelium	Hyperautofluorescence corresponding to areas of orange pigment	Retinal disruptions, detachments, debris, and hyperreflective foci	Deep areas of microcirculation and smooth, well-demarked areas of hypocyanescence in the late phase
Lymphoma	Multifocal creamy-yellow patches at the level of the choroid often with subretinal fluid, a diffuse distribution, ill-defined margins, and a lack of intrinsic pigmentation	Acoustically hollow thickening of the choroid with areas of posterior epibulbar extensions (B-scan)	Granular (leopard spot pattern) appearance and hypofluorescence in the early to mid-phases	Areas of hyperautofluorescence	Segments of the retinal pigment epithelium that are nodular, elevated, hyperreflective, or detached	Clusters of small hypocyanescent lesions

Due to its rich vascular supply, the choroid is the most common ocular structure affected by metastasis and the reported mean survival time following a diagnosis of ocular metastasis is 21 months [37, 38]. The most common originating sites of distant metastasis to the choroid include the breast (53%), lungs (20%), and GI tract (4%) [39]. Patients often complain of painless blurry vision but may also note flashes and floaters, pain, or be asymptomatic [37, 39–42]. Choroidal metastases can be identified on fundoscopy as a yellow subretinal mass located posterior to the equator with subretinal fluid [37, 39]. There are also documented cases of alternative presentations including an orange mass (associated with renal cell carcinoma, carcinoid tumors, thyroid cancers) and brown-gray lesions (associated with metastatic melanoma) [37, 39]. Several imaging modalities can be utilized to aid in the diagnosis of choroidal metastasis. AF commonly identifies subretinal fluid and lipofuscin (scattered clumps of brown pigment) as areas of hyperautofluorescence, while FA demonstrates early hypofluorescence in the arterial phase and late hyperfluorescence in the venous phase [43–46]. On ultrasound, choroidal metastases appear as echo-dense lesions (B-scan) with high internal reflectivity (A-scan), as opposed to choroidal melanomas which appear as acoustically hollow lesions (B-scan) with low internal reflectivity (A-scan) [37, 47–49]. Additionally, OCT’s may be notable for an undulating surface overlying the metastasis and thickening of the retinal pigment along areas of subretinal fluid [50], and ICG’s typically demonstrate hypocyanescence at all stages [51, 52].

It is also important to consider choroidal nevi and amelanotic choroidal melanomas (ACM) in the differential for a choroidal granuloma. Choroidal nevi are benign tumors of the posterior pole with a reported incidence of 6.5–33% [53, 54]. Of those nevi, 5–6% can be further classified as amelanotic [53, 54]. Choroidal nevi can be identified on fundoscopy as flat, or slightly elevated lesions ranging from slate-gray to an amelanotic yellow white color, with poorly defined margins and often associated with drusen [53–55]. On the other hand, ACMs are malignant tumors of the uvea and while rare, are the most common malignant primary intraocular tumor [56–58]. They present with prominent vascularity, an accumulation of subretinal fluid, a serous retinal detachment, and lipofuscin with an orange or golden-brown appearance [53, 57, 59]. OCT can be used to identify the presence of retinal disruptions, detachments, debris, and hyperreflective foci [49, 57]. These lesions typically display hyperautofluorescence corresponding to areas of orange pigment on AF [49, 60] and on FA may demonstrate signs of tumor growth such as double circulation, extensive leakage with progressive fluorescence, late staining of the lesions, and multiple areas of pinpoint leaks affecting the retinal pigment epithelium [49, 53, 57]. Additionally, ICG’s can be used to identify deeper areas of microcirculation and smooth, well-demarked areas of hypocyanescence in the late phase [49, 57].

Uveal lymphoma may present similarly to choroidal granulomas with many patients being asymptomatic or having a slow progression of symptoms [61–65]. They can be identified on fundoscopy as multifocal creamy-yellow patches at the level of the choroid often with subretinal fluid, a diffuse distribution, ill-defined margins, and a lack of intrinsic pigmentation [61–64]. It is best identified using ultrasonography and on B-scan, often appears as an acoustically hollow thickening of the choroid with areas of posterior epibulbar extensions [61–63]. On FA, they may present as areas with a granular (leopard spot pattern) appearance and hypofluorescence in the early to mid-phases [66, 67]. Additionally, these granular regions may appear as areas of hyperautofluorescence on AF [66, 67], or as clusters of small hypocyanescent lesions on ICG [68]. OCT’s can also be used to identify segments of the retinal pigment epithelium that are nodular, elevated, hyperreflective, or detached [66, 67].

Although extensive research is being conducted, the etiology of sarcoidosis remains unclear [69]. The current evidence suggests that an unknown antigen triggers an aberrant immune response in a genetically susceptible individual, however, none of the investigated antigens have been significant yet [69]. In the cases of Ung et al. [16] and Doycheva et al. [18], immunotherapy agents (Nivolumab and INF-a) had been initiated prior to the development of choroidal granulomas. In both cases patients were weaned off their agents, but only in Doycheva were they started on steroid therapy. Ung et al. reported that their patient was asymptomatic at presentation and upon discontinuation of Nivolumab the size of the granuloma stabilized and did not display progression up to the 1 year follow-up [16]. Doycheva et al. described three separate cases of patients with disrupted vision while on INF-a for chronic hepatitis C. In all three cases, INF-a was tapered, and topical steroids were introduced, but only one case was given systemic steroids. They observed resolution of the mass and improvement in the visual acuity of all three patients [18].

It is imperative to consider the full differential diagnosis when evaluating a choroidal lesion as the treatment plan will differ significantly depending on the final diagnosis. If there is ever a time where inconsistency between symptoms, fundoscopy, and imaging arises, or there is doubt, a choroidal sarcoid granuloma should be considered. A choroidal granuloma has a wide variety of clinical presentations and can affect both sexes, several racial groups, and a broad age range. It is not always preceded by a diagnosis of sarcoidosis and treatment with corticosteroids is often curative. Therefore, regardless of the characteristics of the patient, it should always be included when considering a differential for a choroidal lesion.

## Conclusion

This review summarizes the 26 cases of choroidal sarcoid granuloma in the English language and presents a unique case of a sarcoid granuloma initially mistaken for a choroidal neoplasm. Choroidal granulomas are a rare presentation of ocular sarcoidosis that can impede vision. They are typically yellow lesions, single or multiple, found temporal to or at the macula. Primary testing including fundoscopy, fluorescein angiography, fundus autofluorescence, A/B-scan, and OCT are useful for diagnosing and monitoring response to treatment in patients with a choroidal granuloma. Systemic work-up and ACE levels are helpful in diagnosing atypical choroidal lesions in the absence of a confirmed sarcoidosis diagnosis. Steroids are a mainstay of treatment for sarcoidosis and are effective at treating choroidal granulomas. Early recognition and diagnosis of choroidal granulomas is imperative as treatment can be curative and sight-sparing.

## Data Availability

All data generated or analyzed during this study are included in this published article.

## Abbreviations

ACM: Amelanotic choroidal melanomas
AF: Fundus autofluorescence
FA: Fluorescein angiography
FDG: Fluorodeoxyglucose
ICG: Indocyanine green
INF-a: Interferon-alpha
OCT: Optical coherence tomography
PET: Positron emission tomography
SRF: Subretinal Fluid
